# Staged management of a large ischemic heel ulcer in a diabetes patient: a case report

**DOI:** 10.3389/fendo.2023.1198818

**Published:** 2023-06-16

**Authors:** Yuedong Chen, Hui Yang, Wei Wang, Yinchen Chen, Dong Jiang, Yihui Li, Liyi Li, Wengbo Yang, Aiping Wang

**Affiliations:** ^1^Diabetic Foot Center, Nanjing Junxie Hospital, Nanjing, Jiangsu, China; ^2^Department of Orthopaedics, Nanjing First Hospital, Nanjing, Jiangsu, China

**Keywords:** heel ulcer, ischemic, vascular reconstruction, peroneal artery perforator flap, staged

## Abstract

Heel ulcer is one of the severe complications of patients with diabetes mellitus, which poses a high risk for foot infection and amputation, especially in patients with peripheral arterial disease and neuropathy. Researchers have searched for new treatments for treating diabetic foot ulcers in recent years. In this case report, we demonstrated the treatment of large ischemic ulcers for the first time in a diabetic patient. The overall treatment goal of this patient was designed to improve blood supply to her diseased lower extremities and close the ulcer. This two-stage reconstruction approach resulted in an ulcer-free, stable, plantigrade foot at postoperative follow-up.

## 1 Introduction

Diabetes mellitus (DM) is a chronic, demanding metabolic disease that affects individuals, their families, and society worldwide (1). Approximately 463 million people suffer from DM in the world, and this number is expected to rise by 25% by 2030 (2). Among all complications, diabetic foot ulcers (DFUs) are one of the serious and refractory complications of DM. The latest definition of DFUs refers to infection, ulcer, or destruction of foot tissue caused by lower limb neuropathy and/or peripheral arterial disease in patients with DM (3). Studies have found that the prevalence rate of diabetic foot is 4%–10%, the annual population-based incidence rate is 1.0%–4.1%, and the lifetime incidence rate of diabetic patients may be as high as 25%, which brings a heavy economic burden to patients and their families (4). Patients suffering from DM account for almost 60% of all whole-limb amputations (5). Among these, heel ulcer is one of the severe complications of patients with diabetes, which poses a high risk for foot infection and amputation, especially in patients with peripheral arterial disease (PAD). In this case report, we demonstrated the treatment of large ischemic ulcers for the first time in a diabetic patient.

## 2 Case presentation

### 2.1 The case

A 59-year-old female patient with a 17-year history of DM was transferred to our Diabetic Foot Center due to a non-healing heel ulcer after 2 months of scalding. In the early stage of the disease, a large blister burst with much light-yellow exudation on the skin of the lesion site. Further, the amount of exudate decreased gradually, and the ulcer became black and failed to heal even with some conventional treatments such as dressing change and using antibiotics prescribed by another hospital.

### 2.2 Physical examination

**Figure 1:**
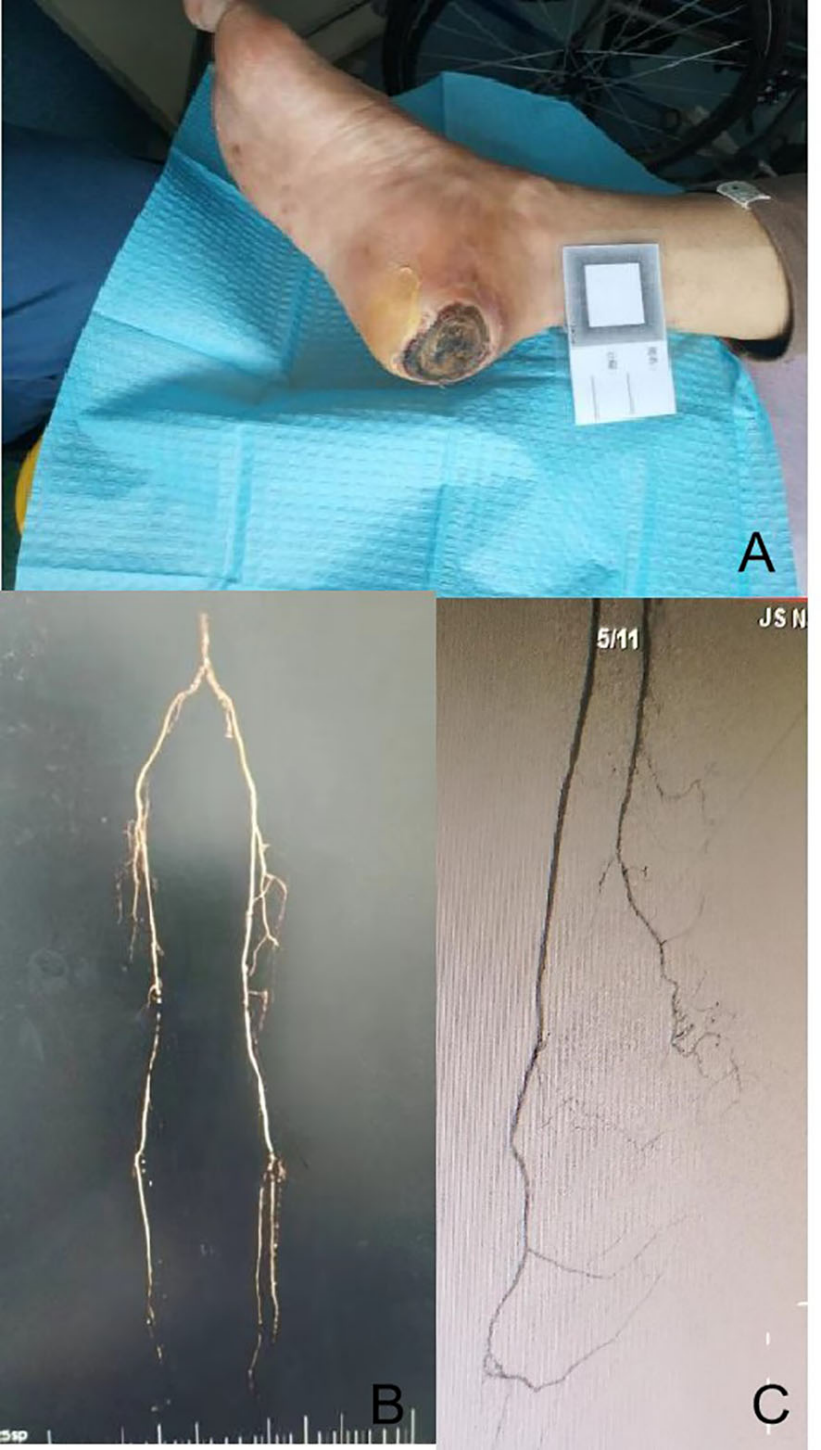
Physical examination of the wound, preoperative CTA examination of lower limb vessels, and angiography after lower limb arterial reconstruction. It was a round ulcer of approximately 5 cm × 5 cm in diameter, which was covered with large black scabs, and the tissue around it was cool, dark red, and swollen **(A)**. Long segment occlusion of the inferior segment of the right superficial femoral artery with segmentary severe stenosis **(B)**. The vascular lumen of the superficial femoral artery, popliteal artery, anterior tibial artery, peroneal artery, and some arterial arches of the foot were unobstructed in the affected limb after lower limb arterial reconstruction **(C)**. CTA, computed tomography angiography.

On both of her feet, the skin temperature was low, and the pulse of the dorsalis pedis artery and posterior tibial artery could not be palpated. There was a large, round ulcer of approximately 5 cm × 5 cm in diameter and unclear in depth. Its surface was covered with large black scabs with a little yellowish exudation visible around it. The tissue around it was cool, dark red, and swollen (**Figure 1A**). Laboratory tests showed her peripheral blood white blood cell (WBC) was 9.1 * 10^9^/L, of which neutrophils accounted for 79.9% and lymphocytes for 12.2%. Meanwhile, the erythrocyte sedimentation rate (ESR) was 79 mm/h, C-reactive protein (CRP) was 12 mg/dl, and procalcitonin (PCT) was 0.41 ng/ml. MRI of the diseased foot revealed local osteomyelitis on the calcaneal surface. B-scan ultrasound of her lower limb vessels showed diffuse moderate-to-severe stenosis in the middle and lower segments of the bilateral femoral artery. Computed tomography angiography (CTA) examination further indicated mild-to-severe stenosis in multiple segments of her bilateral femoral and popliteal arteries, as well as complete occlusion of her right femoral artery and posterior tibial artery (**Figure 1B**).

### 2.3 Diagnosis

There are more than 10 classification methods for diabetic foot, such as Meggitt–Wagner, Texas, PEDIS, SINBAD, and WIfI. The contents and indications of these classification methods are different, and each of them has its own advantages and disadvantages (6, 7). Among them, the WIfI classification has been proposed for the threatened lower limb, based on the three main factors that have an impact on limb amputation risk: wound (W), ischemia (I), and foot infection (“fI”). This classification is commonly used in patients with ischemic diabetic foot, with the advantage of clear evaluation indicators that can help guide the diagnosis and treatment of DFUs, but with the disadvantage of lacking evaluation of diabetic neuropathy. The patient in this case report was evaluated and diagnosed using the WIfI classification. The person in this case report was found to have a large ulcer on the heel with approximately 25 cm^2^ in area, accompanied by severe limb ischemia and heel osteomyelitis by MRI but no systemic inflammatory response syndrome (SIRS). Therefore, the DFUs of this patient were rated as diabetic foot W3 I3 fI2.

### 2.4 Treatment

**Figure 2:**
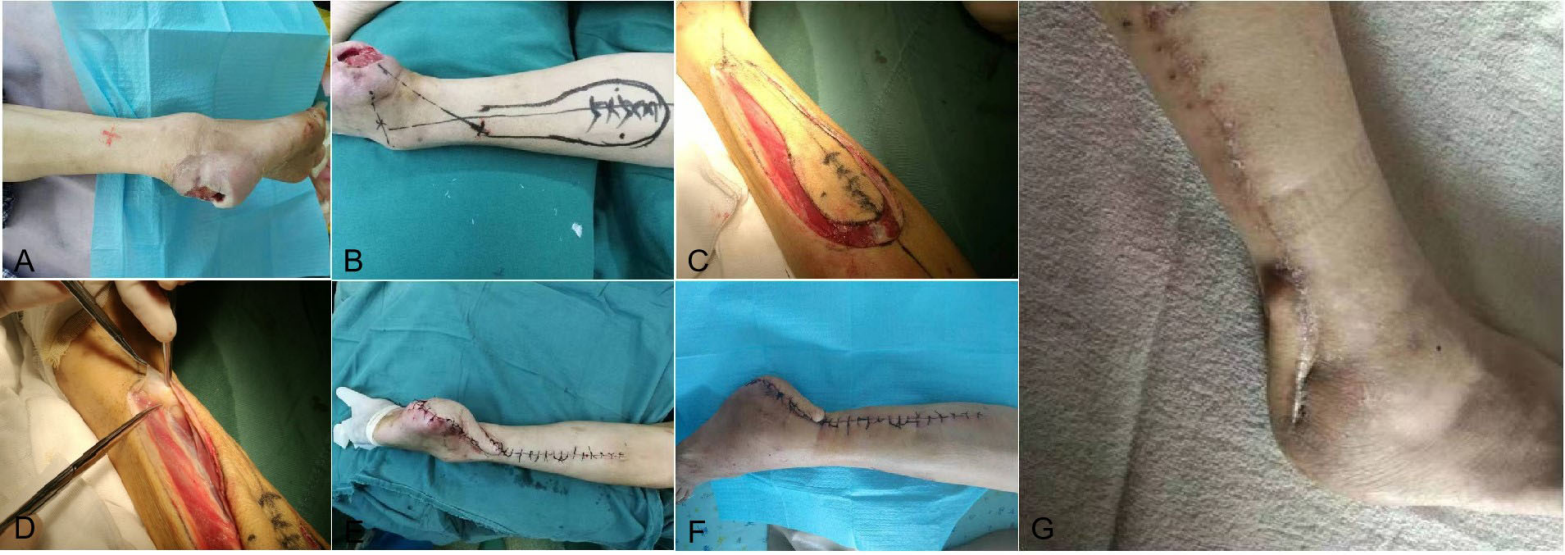
The repair of the wound and postoperative follow-up. After the blood supply improved, the wound repair procedure began. Wound debridement began approximately 4 weeks after the vascular intervention, with fresh granulation tissue hyperplasia at the base of the wound and bleeding after scraping **(A)**. The location of perforating artery of peroneal artery perforator flap **(B)**. Peroneal artery perforator flap design during operation **(C, D)**. The wound condition after the operation **(E)** and dressing change of the wound post-operation **(F)**. At the postoperative follow-up, the patient’s wound healed completely approximately 7 weeks after the operation **(G)**.

First, the surgery of balloon dilation under local anesthesia was planned for this patient to revascularize her right superficial femoral artery and peroneal artery. Preoperative angiography confirmed long segment occlusion of the inferior segment of her right superficial femoral artery with segmentary severe stenosis (**Figure 1B**). Intraoperatively, the superficial femoral artery, popliteal artery, anterior tibial artery, and peroneal artery were successfully revascularized, but the posterior tibial artery was not successfully re-opened. Postoperative angiography showed that the vascular lumen of the superficial femoral artery, popliteal artery, anterior tibial artery, peroneal artery, and some arterial arches of the foot were unobstructed in the affected limb (**Figure 1C**). Subsequently, the patient was then allowed several weeks for the recovery of local blood supply to the wound, while the wound was periodically debrided and dressed in a small range. In the fourth week after surgery, the peroneal perforator flap was transferred and repaired under general anesthesia. Postoperative nursing was also very important. For postoperative nursing, we took the following measures: psychological counseling, keeping the non-weight-bearing state of the limb, keeping the limb warm, raising the limb, and closely observing the blood transport and the color change of the flap. In the 7th week, stitches were removed, and the flap was alive (**Figure 2G**). At the 12th week, the affected limb could be loaded with moderate weight bearing. In the 24th week, the patient was able to walk freely with this limb in which the flap appearance and texture were perfect.

## 3 Discussion

**Figure 3:**
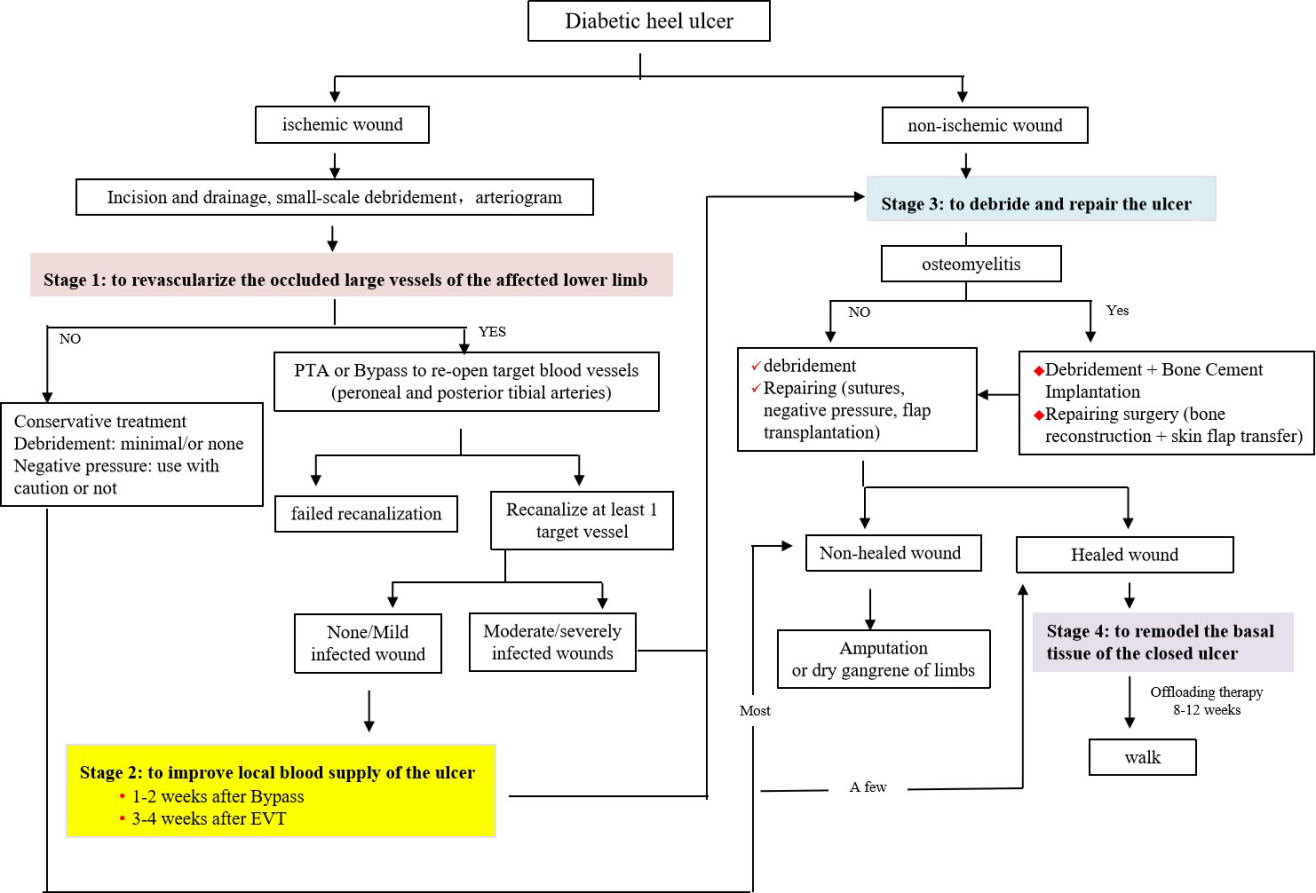
Treatment procedures for heel ulcers. For ischemic diabetic heel ulcer, treatment procedures were implemented in four stages: i) revascularize the occluded large vessels of the affected lower limb, ii) improve the local blood supply of the ulcer, iii) debride and repair the ulcer, and iv) remodel the closed ulcer.

Heel ulcer and ischemia are both predisposing factors for major amputation in diabetic foot patients, and the combination of these two factors increases the risk of this amputation to 20%, which is much higher than that of non-heel ulcer (6.9%) or non-ischemia ulcer (<5.1%) (8). In this case, the overall treatment goal was designed to improve blood supply to her diseased lower extremities and close the ulcer. First of all, we developed a set of treatment procedures for these patients (**Figure 3**), which were implemented in four stages: to revascularize the occluded large vessels of the affected lower limb, to improve the local blood supply of the ulcer, to debride and repair the ulcer, and to remodel the closed ulcer. If the target vessels leading to the heel, namely, the posterior tibial and peroneal arteries, are successfully reconstructed, which could improve the local blood supply, the overall survival rate of the free flap grafted to this site can reach 73%, and the limb salvage rate of the patient can be greatly increased (9, 10).

Under the guidance of the concept of “angiosome”, wound-oriented revascularization surgery is very important for ulcer healing and limb rescue (11–13). In this patient, the ulcer was located in the middle posterior aspect of her right heel, and the preoperative angiography confirmed long segment occlusion of the inferior segment of her right superficial femoral artery with segmentary severe stenosis. The posterior tibial artery and peroneal artery of her right lower extremity should be opened theoretically. However, as shown in **Figure 1C**, the anterior tibial artery, the peroneal artery including the posterior communicating branch, and part arterial arch of the foot were re-opened successfully, but the posterior tibial artery failed to be recanalized. Because the posterior communicating branch of the peroneal artery can compensatively supply blood to the posteromedial heel of the heel, thereby overcoming the adverse effects of the unsuccessful recanalization of her posterior tibial artery, it was believed that the patient still has the possibility of further complex surgeries to repair the wound at this time.

Studies have shown that as skin oxygen tension increases and carbon dioxide pressure decreases progressively for several weeks after a successful percutaneous transluminal angioplasty (PTA), the likelihood of ulcer healing increases dramatically. Therefore, the best time for surgical wound management should be 3–4 weeks after successful PTA revascularization and 1–2 weeks after bypass revascularization (14, 15). Note that during this period, local infection of the wound may worsen following the gradual recovery of blood flow, which needs much more observation and even debridement in advance if necessary. This patient experienced a waiting period of approximately 4 weeks after the vascular intervention, until the blood supply to the injured heel was restored, and the granulation tissue at the base of the wound was bright red and bleeding after scraping.

The following repair process for diabetic foot wounds is a combination of a series of medical methods, which is complicated. As a core component of traditional DFU treatment, debriding the wound not only removes inactive bone or soft tissue that may pose a risk of bacterial colonization and infection but also effectively repairs the wound to the acute stage or hemostasis/coagulation stage, which is conducive to its later healing (16). Some appropriate methods from the four-layer “pyramid” techniques of repairing soft tissue include negative pressure wound therapy/primary closure, local random flaps/skin grafting/bioengineered tissue alternatives, pedicle flaps/local muscle flaps, and free tissue transfer to cover the ulcer (17). Among them, pedicle flaps/local muscle flaps and free tissue transfer are the most difficult and risky and have high technical requirements for the operator, but they are still increasingly used in the treatment of diabetic foot ulcers because of their high effectiveness (18). With the restoration of local blood supply to the heel by recanalizing the peroneal artery successfully, the heel ulcer of this patient was cleaned clearly and closed with a highly challenging ultra-microsurgical peroneal artery perforator flap completely (**Figure 2**).

It is generally known that wound healing is the beginning of its basal tissue remodeling phase. During the healing process of diabetic foot wounds, irregular or irregularly arranged collagen fibers are gradually replaced by newly synthesized, more regular, and elastic collagen fibers until the original scar tissue on the wound becomes soft, has a lighter color, and becomes smooth and orderly. The entire wound remodeling process can last for months or even years until the tensile strength of the tissue within the wound is restored as much as possible. We followed up the patient approximately 7 weeks after the operation, and the wound was completely healed (**Figure 2G**). Moreover, offloading treatment at the wound-healing stage is very important for a DFU patient (19). The female patient was given adequate offloading therapy, such as non-weight bearing for up to 8 weeks after flap transplantation. Ultimately, the patient was able to walk gently with a well-healed flap at the 12th week and with full weight bearing at the 24th week.

## 4 Conclusions

**Figure 4:**
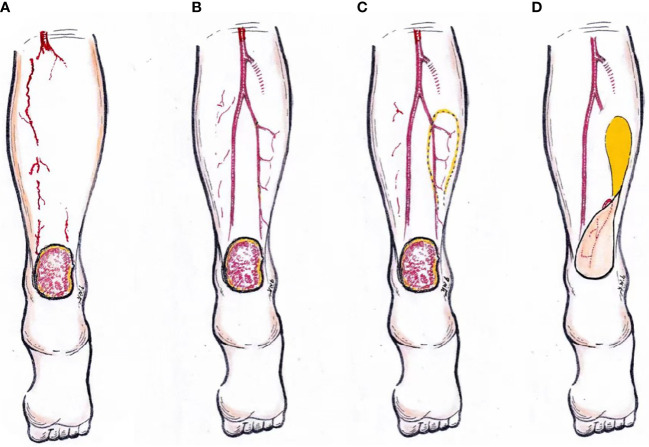
A schematic representation of the surgical management of ischemic diabetic heel ulcer. Assessment of the lower limb blood supply **(A)**. Reconstruction of the target artery vessels **(B)**. Design and operation of peroneal artery perforator flap transfer surgery **(C, D)**.

This case report demonstrates that the management of large ischemic heel ulcers in diabetic patients has its particularity. The diagnosis of diabetic foot requires not only evaluation for ulceration, ischemia, and infection but also differentiation from other causes of heel ulcers. Subsequently, this type of ulcer should be treated in stages according to a certain sequence, as shown in **Figure 4**, to assess and revascularize the occluded large vessels of the affected lower limb, to improve the local blood supply of the ulcer, to debride and repair the ulcer, and finally to remodel the closed ulcer using flap surgery. Among them, the opening of at least one target blood vessel in the heel is the premise, the successful transplantation of the skin flap to cover the wound is the key, and offloading measures such as walking without weight-bearing are also very important in the later stage. In conclusion, as one of the risk factors of major amputation, massive ischemic heel ulcer needs to be carefully diagnosed and treated according to a special procedure.

## Data availability statement

The raw data supporting the conclusions of this article will be made available by the authors, without undue reservation.

## Ethics statement

Written informed consent was obtained from the patient for publication of this report. Written informed consent was obtained from the individual(s) for the publication of any identifiable images or data included in this article.

## Author contributions

YDC was responsible for study conception and design. HY wrote the first draft of the manuscript. YCC, AP edited the manuscript. WW, DJ, LL, WY reviewed the last version of the manuscript. All authors contributed to the article and approved the submitted version
